# Peptide Mediated Antimicrobial Dental Adhesive System

**DOI:** 10.3390/app9030557

**Published:** 2019-02-08

**Authors:** Sheng-Xue Xie, Kyle Boone, Sarah Kay VanOosten, Esra Yuca, Linyong Song, Xueping Ge, Qiang Ye, Paulette Spencer, Candan Tamerler

**Affiliations:** 1 Institute for Bioengineering Research, University of Kansas, 1530 W. 15th St., Lawrence, KS 66045, USA; 2 Bioengineering Program, 1530 W. 15th St., University of Kansas, Lawrence, KS 66045, USA; 3 Department of Molecular Biology and Genetics, Yildiz Technical University, 34210 Istanbul, Turkey; 4 Department of Mechanical Engineering, 1530 W. 15th St., University of Kansas, Lawrence, KS 66045, USA

**Keywords:** antimicrobial peptide, polylysine, adhesive formulation, hybrid interface, dental composites

## Abstract

The most common cause for dental composite failures is secondary caries due to invasive bacterial colonization of the adhesive/dentin (a/d) interface. Innate material weakness often lead to an insufficient seal between the adhesive and dentin. Consequently, bacterial by-products invade the porous a/d interface leading to material degradation and dental caries. Current approaches to achieve antibacterial properties in these materials continue to raise concerns regarding hypersensitivity and antibiotic resistance. Herein, we have developed a multi-faceted, bio-functionalized approach to overcome the vulnerability of such interfaces. An antimicrobial adhesive formulation was designed using a combination of antimicrobial peptide and a ε-polylysine resin system. Effector molecules boasting innate immunity are brought together with a biopolymer offering a two-fold biomimetic design approach. The selection of ε-polylysine was inspired due to its non-toxic nature and common use as food preservative. Biomolecular characterization and functional activity of our engineered dental adhesive formulation were assessed and the combinatorial formulation demonstrated significant antimicrobial activity against *Streptococcus mutans.* Our antimicrobial peptide-hydrophilic adhesive hybrid system design offers advanced, biofunctional properties at the critical a/d interface.

## 1. Introduction

Despite the significant increase in the usage of composite materials in restorative dentistry, their reduced average lifetime remains a major concern [1]. Clinical data suggest that over half of all restorative operations performed in contemporary dentistry are procedures aimed to replace or repair regions at which previous restorations have failed [2]. These failures are primarily attributed to recurrent caries and infection [3]. The majority of composite restoration failures across all patient groups are linked to the local infiltration and colonization of harmful bacteria. Invasive bacteria can infiltrate weak or impaired regions at the adhesive-dentin (a/d) interface leading to further biodegradation and secondary loss of adhesion, microleakage, and decay [4]. This vulnerable interface has been identified as the weakest link for the reduced durability of modern composite restorations [2,4–14]. Therefore, it is critical to design novel, enhanced composite adhesive formulations aimed to achieve improved interfacial binding and provide protection against bacterial colonization at the susceptible a/d interface.

Various approaches have been investigated to improve the longevity of restorative composite. In our recent study, lysine incorporation into a dental adhesive system similar to a HEMA/Bis-GMA based commercial analog resulted in neutralization. Modulating the pH of the dental adhesive by adding a single amino acid, lysine, can lead to overcome the adverse effects of the acidic microenvironment and enhance the durability of composite restorations [15]. Other approaches explored to incorporate antimicrobial agents, and consecutively develop antimicrobial properties in the restorative composites. These approaches include the use of zinc- or silver-based nanoparticles with innate antimicrobial properties through metallic ion release [16–18]. Other techniques have relied on the use of quaternary ammonium compounds (QACs) chemically bound to (immobilized) or copolymerized with the composite monomer formulation [18–21]. Additionally, several studies have designed composite materials which release low-molecular weight antibacterial agents such as chlorhexidine (CHX) [20]. Although many groups have published promising data using these approaches, limitations have also been reported. Determining appropriate nanoparticle size and concentration as well as an optimal technique for stabilization or immobilization and incorporation within the adhesive resin has been difficult. Additionally, QAC-related antimicrobial resistance has been shown to occur at sub-inhibitory concentrations through both intrinsic and acquired resistance mechanisms [22–24]. Though CHX is recognized as the most effective chemical antiplaque agent [25–28], its limitations include adverse effects for taste, mouth discoloration, mucosal irritation, and desquamation of the gums [29,30].

The emergence of drug-resistant bacteria is another major driving force for seeking new antibacterial agents to replace current antibiotics to prevent and fight bacterial infections [31–33]. Antimicrobial peptides, which are naturally integrated in the oral fluids [34–36], are a promising solution to prevent bacterial infections at the a/d interface since they are selective, biodegradable, and can remain active across evolutionary time scales [37–40]. Thus, antimicrobial peptides have been increasingly explored for medical use over the last decade. While antimicrobial peptides may have higher inhibitory concentrations than other agents, they also have less mammalian cellular toxicity, no staining effect, and minimal observed bacterial resistance rates [41]. The efficacy of antimicrobial peptides is postulated to be dependent upon their secondary structure characteristics that reveal the pattern of hydrogen bonding in the peptide backbone [42]. Our group has previously evaluated the secondary structure features of antimicrobial peptides through circular dichroism [43,44]. This technique uses the preferential absorption of left-handed or right-handed elliptically polarized light to observe changes in the peptide backbone. Apparent differences in the circular dichroism spectra may indicate functional changes of the peptides, owing to the conditional harmony of structure–function relationships [45]. Due to its advantageous properties, antimicrobial peptides have been used for various applications in the pharmaceutical and food industries [46]. Significant progress has been made in recent years on the control of oral pathogens, such as *S. mutans*, with antimicrobial peptides [47]. Currently, antimicrobial peptides (AMPs), derived from both natural and synthetic sources, have shown inhibition of *S. mutans* [48,49]. AMPs have been used for various dental application research. Aida, et al., 2018 have developed an antimicrobial peptide loaded drug delivery system to prevent dental caries [50]. AMP has also been showed to reduce periodontal pathogens [51]. Antimicrobial peptides have also been incorporated in dental titanium implants [52,53]. GL13K, a derivative of an oral peptide, inhibited bacterial activity on the titanium surface when immobilized through covalent linkages. Our group explored antimicrobial peptides to inhibit the activity of bacteria on the titanium surface using a solid-binding peptide based non-covalent immobilization strategy. Over the last decade, we have identified and designed a wide range of solid material binding peptides and utilized these peptides by coupling with diverse bioactivities as chimeric biomolecules. The use of solid-binding peptides enabled the chimeric peptides self assemble onto different material surfaces and resulted in enhanced variety of bioactivities including antibacterial, cell targeting, and signaling to guide cell fate [43,44,54–57]. In a recent study, nisin, an antimicrobial peptide, was used to make a cured nisin-incorporated dental adhesive that exhibited a significant inhibitory effect on the growth of *S. mutans*. The inhibitory effect was strengthened as the nisin concentration increased however, there were no significant differences in the agar diffusion test for the cured nisin-incorporated adhesives when compared to the control group [58,59].

ε-Polylysine (ε-pL) is already recognized for its safe use as a commercial food preservative due to its natural antimicrobial properties [60,61]. As a small homopolymer, ε-pL, was approved by the FDA as a food additive, noting its antimicrobial activity and low toxicity to human cells [62]. Panpisut and colleagues recently explored polylysine addition to the mixture of monocalcium phosphate monohydrate and tristrontium phosphate into a dental composite system and demonstrated increased apatite layer thickness [63].

Herein, we propose a novel, bio-enabled dental adhesive system with multiple antibacterial mechanisms and innate biocompatibility to address the critical weakness of bacteria-induced degradation at the a/d interface. We explore the effects of adding ε-pL to our dental adhesive formulation and incorporating antimicrobial peptide to the adhesive system as part of our approach to provide multiple antibacterial mechanisms. First, we developed an amine-functionalized dental adhesive formulation, building upon our previous work which addressed the challenges of the hydration difference between the dental adhesive and the demineralized dentin [64–66]. Next, we added ε-pL to the adhesive formulation coupled with antimicrobial peptides as an additional mechanism to combat bacterial colonization at the a/d interface. To achieve this goal, we engineered derivatives of an established antimicrobial peptide, GH12, with known activity against *S. mutans* [67]. GH12 has been shown to reduce the cariogenic virulence factors of *S. mutans*, an activity that makes GH12 a candidate for dental caries prevention [68]. In addition, GH12 was successfully employed in a rodent model in a pioneer study aimed to analyze the efficacy of an antimicrobial peptide on the onset and development of dental caries [69]. Promising sequence derivatives were selected for synthesis through computational structure generation verified via analysis of GH12 and modified peptide folding using circular dichroism in the presence of a kosmotropic agent. To ensure effective activity, the peptide was designed to allow for amine coupling to the enhanced dental adhesive formulation through the addition of a spacer sequence. Our results demonstrate that the addition of both ε-pL and the engineered antimicrobial peptide components provide critical bacterial inhibition for the adhesive formulations.

## 2. Materials and Methods

### 2.1. Materials

Chlorhexidine digluconate (CHX) was from Sigma-Aldrich. BD™ Bacto™ brain heart infusion, BD BBL dehydrated brain heart infusion agar, and Corning™ Clear polystyrene 96-well microplates, Corning™ 3370 were obtained from Fisher. AlamarBlue assay kit was obtained from Fisher.

Triethylene glycol dimethacrylate (TEGDMA), 2-hydroxyethylmethacrylate (HEMA) and 2-methacryloyloxyethyl phosphorylcholine (MPC) were used as received (Sigma-Aldrich) without further purification. Camphorquinone (CQ), ethyl-4-(dimethylamino) benzoate (EDMAB) and diphenyliodonium hexafluorophosphate (DPIHP) were obtained from Sigma-Aldrich (St. Louis, MO, USA) and used as a three-component-photoinitiator system without further purification. ε-pL hydrochloride was purchased from Bonding Chemical (Katy, TX, USA). To obtain ε-pL), 10 g ε-pL hydrochloride was dissolved in 60 mL sodium bicarbonate buffer (NaHCO3, 1.5M) and dialyzed with membrane (Spectra/Por®7 Dialysis Tubing, MWCO 1kD) in deionized water for 4 days. Then the solution was dried at 37 °C and ε-pL was obtained.

### 2.2. Peptide Synthesis and Purification

The antimicrobial peptides: GH12 (GLLWHLLHHLLH), GH12-M1 (K_GGGSG_ GLLWHLLHHLLH) and GH12-M2 (GLLWHLLHHLLH_GSGGG_K) were synthesized by the solid-phase peptide synthesizer (AAPPTEC Focus XC) through Fmoc-chemistry. Crude peptides were first cleaved from the resin and then purified by reversed-phase HPLC with Luna 10 μm C18 (2) 100 A, 250 *×* 4.6 mm column. HPLC fractions were analyzed for purity by analytical HPLC with Luna 5 μm C18 (2) 100 A, 250 *×* 4.6 mm column. The purified peptide fractions were combined, lyophilized and stored at *−*20 °C. The amino acid sequence of the peptides was confirmed by electrospray ionization mass spectrometry using a Qtof Premier (Waters Corp., Milford, MA, USA) instrument. Sequence confirming fragment ions were generated by collision-induced dissociation of peptide doubly charged ions in the collision cell and mass-measured in the time-of-flight analyzer. Peptides were then desalted and resolved from other components in the sample by RP HPLC on C18 media developed with a water-CH_3_CN gradient and effluent delivered to the ESI source of the Qtof. Peptide solutions were prepared fresh from stock powders for each experiment.

### 2.3. Streptococcus mutans UA159 Culture

Antimicrobial activity was assessed in an *S. mutans* UA159 bacterial strain (American Type Culture Collection (ATCC 700610)). Following ATCC standard protocol, the lyophilized bacteria were reconstituted in BHI medium (BD Difco) and streaked onto prepared BHI agar (BD Difco). The stock culture plate was incubated for 24 h at 37 °C with 5% carbon dioxide (CO_2_). Overnight cultures of *S. mutans* UA159 were prepared fresh, prior to each experiment, by inoculating 10 mL of fresh BHI medium with a single colony selected from the stock culture plate and incubated for 16 h. The overnight cultures were diluted in fresh BHI media and growth was monitored via optical density measurements at 600 nm (OD_600_) on a Cytation3 multimodal plate reader (BioTek) [17].

### 2.4. Inhibitory Concentration (IC_50_) Assays

Inhibitory concentration assays were conducted using a standard broth microdilution method, according to Clinical and Laboratory Standards Institute (CLSI) protocol [70]. Briefly, two-fold serial dilutions of compounds were prepared with sterile deionized water to achieve concentrations ranging from 5.0 to 2500 μg/mL in a volume of 20.0 μL, after which 80.0 μL of BHI (Brain Heart Infusion) broth and 100.0 μL of bacterial culture containing 1.0 *×* 10^6^ CFU/mL were added. Thus, each well contained final peptide concentrations ranging from 0.5 to 250 μg/ml in a final volume of 200 μL and a final bacterial concentration of 5.0 *×* 10^5^ CFU/mL. Our protocol included chlorhexidine as the positive control for antimicrobial function. As control samples, microwells containing chlorhexidine digluconate (CHX) with concentrations ranging from 0.04 to 63 μg/mL were also combined with BHI broth and bacterial culture. Although 20 μL of H_2_O was used as a negative control, we recognize that the water effect may be creating different interface issues which may lead to slight variability in results. The measurements were measured and recorded according to a blank subtraction. The experimental blank controls consisted of 20 μL of H_2_O and 180 μL of medium without cells. The 96-well plate was incubated in the presence of 5% CO_2_ at 37 °C overnight. The IC_50_ value was defined as the lowest peptide, CHX, and ε-pL concentration corresponding to 50% inhibition of cellular viability measured via AlamarBlue assay.

### 2.5. Preparation of Adhesive Formulations

Adhesive formulations are reported in Table 1 and experiments were conducted to study effects of peptide coupling and antimicrobial activity of either a commercial analogue control or our experimental ε-pL adhesive formulation. The control formulation contained HEMA/TEGDMA/MPC at 64/10/10 wt%, respectively, whereas the experimental formulation reduced the HEMA concentration by 5 wt% to include ε-pL (ε-pL/HEMA/TEGDMA/MPC at 5/59/10/10 wt%, respectively). Water (14 wt%) was added as the solvent to achieve more homogenous monomer solutions. The compositions of the three-component initiator system were CQ (0.5 wt%), EDMAB (0.5 wt%), and DPIHP (1.0 wt%) with respect to the total amount of monomers and solvent. Both the control and experimental resin mixtures were prepared in amber vials and protected from light. The solutions were mixed for 24 h at room temperature to promote complete dissolution and formation of a homogeneous solution.

### 2.6. Disc Specimen Preparation

Disc specimens were prepared by injecting the liquid resins into a standard aluminum hermetic lid (Tzero®, P/N:901600.901) and covering them with a glass cover (22 mm × 30 mm, Fisherfinest®) to prevent oxygen exposure. The specimens were light-cured for 40 s at 23 *±* 2 °C with a commercial visible-light-polymerization unit (Spectrum®, Dentsply, Milford, DE, USA) at an intensity of 550 mW/cm^2^. The polymerized disc specimens were stored, protected from light, at 23 ± 2 °C for at least 48 h. Subsequently, all specimens were prewashed by soaking in water (250 mL) for 5 days to remove any unreacted components prior to antimicrobial assays. Water was changed daily.

### 2.7. Circular Dichroism

Circular dichroism (CD) was used to determine peptide folding in solutions containing 0–90% (vol%) of trifluoroethanol (TFE) to mimic surface interactions. Spectra were recorded with a CD spectrometer (JASCO, J-815) at room temperature, using a 1.0 mm cuvette. Each peptide sample was dissolved at 0.2 mg/mL in 10 mM potassium phosphate containing 100 mM (NH_4_)_2_SO_4_ (pH 7.4) with and without TFE at 4 °C for 16 h. A spectral range was acquired from 185 to 300 nm at a scanning speed of 60 nm/min for each sample and reported values represent an averaged spectrum across triplicate measurements. The secondary structure Far-UV CD spectra were processed with the tools of CD Pro [71]. The mean residue ellipticity of each sample was converted to mean residue absorbance and further processed with CRDATA.exe to create the input file for SELCON3.exe, CDSSTR.exe and CONTILL.exe. For each set of data, the reference set selected was SMP50. The fractions of secondary structure (Regular Helix, Distorted Helix, Regular Sheet, Distorted Sheet, Turns and Unordered) were averaged for all three CD Pro tools (SELCON3, CDSSTR, and CONTILL).

### 2.8. Vibration Spectroscopy

#### 2.8.1. Fourier-Transform Infrared Spectroscopy (FTIR)

The molecular structure of the synthesized antibacterial peptide was confirmed using FTIR (Spectrum 400, Perkin-Elmer, Waltham, MA, USA), equipped with an attenuated total reflectance (ATR) accessory (PIKE Technologies Gladi-ATR, Madison, WI, USA) at a spectral resolution of 4 cm^*−*1^.

#### 2.8.2. Raman Spectroscopy

Lyophilized peptide powders were placed onto a clean microscopic glass slide. The samples were mounted in a computer-controlled, high precision *x*-*y* stage. The Raman spectra were collected by using an integrated fully automated confocal microscope (Horiba Jobin Yvon ARAMIS micro-Raman microscope) equipped with internal laser excitation (HeNe: 632.8nm). The instrument conditions were: 200 μm confocal hole, 150 μm wide entrance slit, 600 gr/mm grating, 20 s exposure time, and 100 *×* objective Olympus lens. Data processing was performed using LabSPEC 5 (HORIBA Jobin Yvon).

### 2.9. Computational Structure Flexibility

To computationally assess peptide structure flexibility, structure decoys were generated with PyRosetta tools using a developed protocol [54]. Briefly, Rosetta server fragments of lengths of 3 and 9 were randomly inserted into a decoy structure [72]. Inserted fragments resulting in a reduction of energy, triggers the program to change the backbone of the decoy. Fragments not indicating energy reduction are accepted with a probability inversely related to the exponential of the increase of the energy. This fragment insertion method uses segments of determined protein structure, often crystallized protein structure to search the peptide structural space. The observed percentage of each secondary structure were calculated by the DSSP program at each peptide residue position [73]. Percentages for each peptide fragment were aggregated for direct comparison with collected CD decomposition values using customized scripts.

### 2.10. Antimicrobial Activity Assays of Resin-disc Diffusion in Solution

In-solution antimicrobial activity of peptide-treated resin discs was assessed. For these studies, each disc was soaked overnight in 100 μL sterile deionized water, peptide, or CHX solution at 4 °C. The soaked discs were rinsed 4 times with 100 μL of H_2_O, with excess H_2_O on the disc surface gently removed by blotting with Kimwipes. Each soaked and rinsed disc was carefully transferred to a microwell of a 96-well plate. 20 μL H_2_O, 80 μL of BHI broth and 100 μL of BHI broth containing 1 *×* 10^6^ CFU/mL *S. mutans* UA159 cells were added to each well. The final bacterial concentration in each well was 5.0 *×* 10^5^ CFU/Ml [74]. *S. mutans* UA159 bacteria without any disc present served as the positive control and the negative control was 20 μL of 63 μg/mL CHX solution instead of H_2_O. The blank well contained 20 μL H_2_O and 180 μL medium without cells. The 96-well plate was incubated in the presence of 5% CO_2_ at 37 °C overnight. The solution of each well was transferred to a new plate and the OD_600_ was recorded by Cytation3.

Absorbance-based in-solution measurements rely on turbidity at OD_600_ and therefore do not provide quantitative delineation between viable and non-viable *S. mutans* UA159 cells. Thus, an alternative fluorescent method, described below, was used to ensure the antimicrobial activity of the system was accurately assessed.

### 2.11. Fluorescence-Based Bacterial Viability Assays

The viability of *S. mutans* bacteria was measured using an AlamarBlue (ThermoFisher Scientific) assay [75]. Cellular activity was measured via fluorescence spectroscopy (Ex/Em 565/595 nm) following the bacteria’s metabolic conversion of resazurin to the fluorescent molecule, resorufin. Following the manufacturer’s protocol, *S. mutans* bacteria were grown overnight in a 96-well plate format. Following mixing the solution via pipette, 5 μL of bacterial culture was transferred to a new plate having 195 μL of fresh BHI broth in each well. Next, 20 μL of AlamarBlue dye (10 %vol.) was carefully added into each well and gently mixed. All samples were allowed to wait for 2 h to properly metabolize the reagent in the presence of 5% CO_2_ at 37°C prior to fluorescent activity measurements.

### 2.12. Statistical Analyses

For all experimental groups, the differences were evaluated using one-way analysis of variance (ANOVA), together with Tukey’s test at *α* = 0.05 to identify significant differences.

## 3. Results and Discussion

### 3.1. Antibacterial Activity of the Peptide

**Figure 1.**
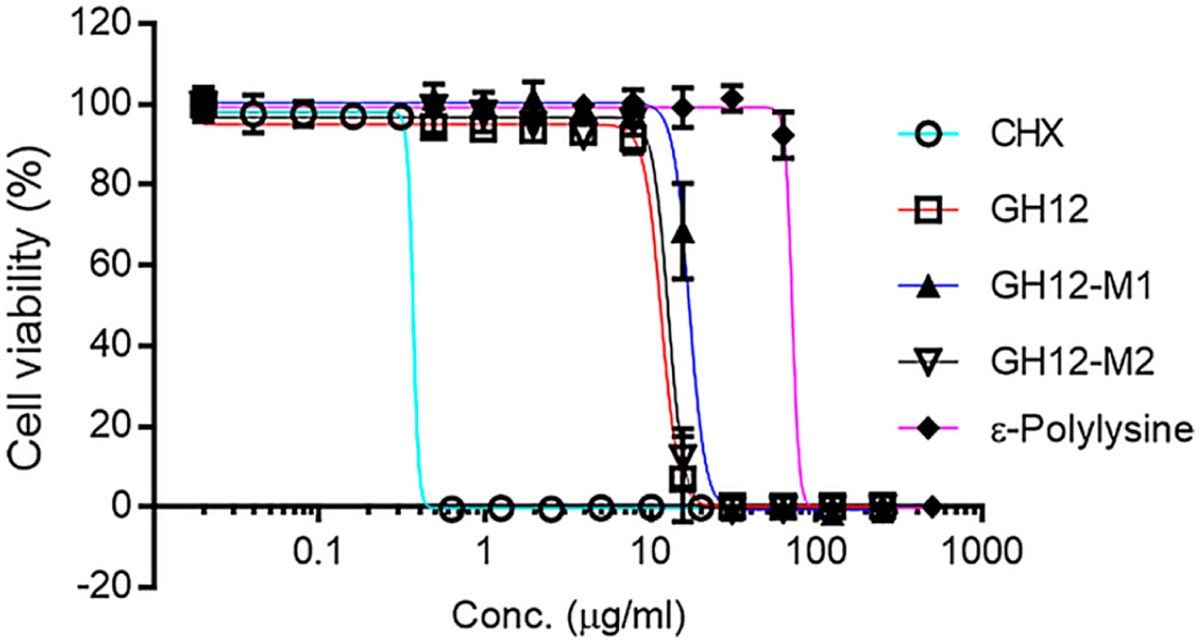
Antibacterial activity of chlorhexidine (CHX), antibacterial peptides and ε-polylysine *S. mutans.*

Table 2 and Figure 1 provide the antimicrobial efficacy of the peptides explored in this study. In addition to the GH12 peptide sequence we explored the sequence modifications for this peptide for effective delivery into the adhesive formulations [67]. We first synthesized a sequence featuring a lysine residue, attached via a spacer sequence [43] then we changed the C-terminal of the peptide to COOH since this would replace the adhesive’s lost carboxy functional groups due to the peptide conjugation. The next design choice was aimed to select the peptide terminus to attach the spacer sequence that would allow for optimal antimicrobial activity. As the control, we used the GH12-NH_2_ (with an C-terminal amide), the reported MIC value of GH12-NH_2_ was about 6.7 μg/mL against *S. mutans* [67]. The GH12 peptide was synthesized to feature a carboxyl group to allow for further sequence modifications involving additional amine-containing moieties such as polypeptide chains or monomers. Next, we designed GH12-M1 to include a spacer domain featuring a single lysine residue at the N-terminal for coupling to the polymer. Additionally, a GH12-M2 peptide was created with the same spacer sequence and single lysine residue as GH12-M1, but placed at the C-terminus of the peptide.

The activity of each peptide was evaluated against *S. mutans* UA159 using an inhibitory concentration (IC_50_) assay. The minimum inhibitory concentration (MIC) values for all synthesized peptides were calculated at 31.7 μg/mL (Figures S1–S5). CHX was used as a positive control and exhibited an IC_50_ value of 0.37 μg/mL. The IC_50_ of GH12, GH12-M2, and GH12-M1 were found 11.6, 12.8, and 17.1 μg/mL, respectively. We next investigated the addition of ε-pL to our formulation which resulted in the IC_50_ value about 71.3 μg/mL. The N-terminal modification (GH12-M1) demonstrated a larger IC_50_ value indicating reduced activity when compared to the unmodified peptide (See Table 2). The spacer modification carried out at the C-terminal (GH12-M2) resulted in a slight increase in the IC_50_ value as compared to the unmodified form. GH12-M2 was therefore selected for further coupling studies due to its superior antimicrobial efficacy as well as flexibility allowing for more direct coupling to an adhesive formulation.

### 3.2. Effect of Peptide Conformation on Function

**Figure 2.**
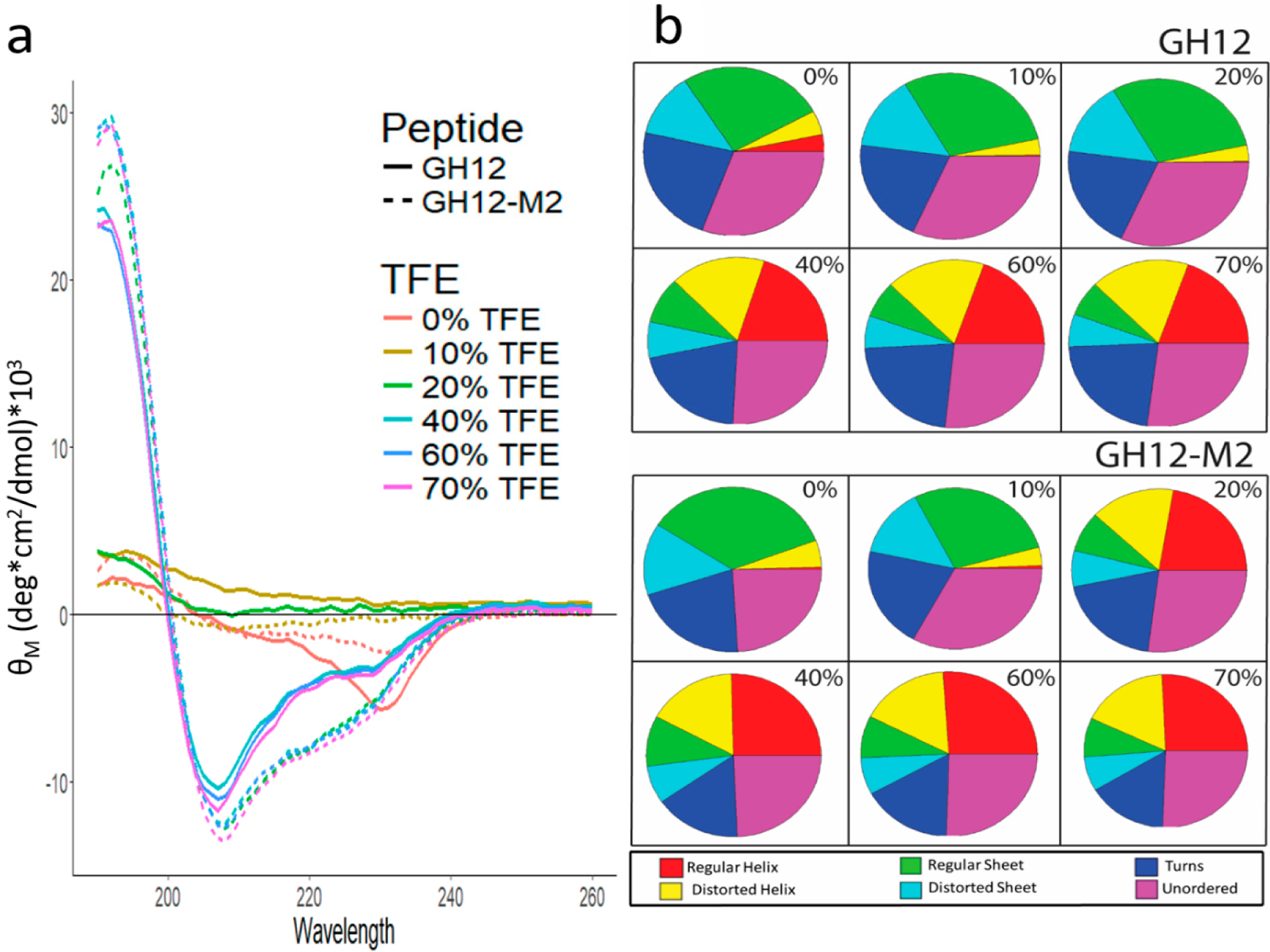
(a) Mean residue ellipticity (MRE) of far-UV CD Spectra for GH12 and GH12-M2 with varying levels of 2,2,2-trifluoroethanol (TFE). (b) Circular dichroism spectra deconvolution through CD Pro analysis. The percentages indicate the amount of TFE in the solution.

Structure-function relations are critical in peptide function, hence we explored the structural differences that may occur between modified GH12-M2 and unmodified GH12 prior to studies on peptide coupling to adhesive formulation. First, we performed circular dichroism spectroscopy to investigate the secondary structure and folding properties of the peptides in solution. We used different buffers to test the conformation of the peptides including a range of concentrations of trifluoroethanol (TFE) to mimic their folding behavior at the solid interfaces. The circular dichroism spectrum was relatively flat for GH12 without TFE in ammonium sulfate and potassium phosphate buffers at pH 7.4 with a negative peak near 230 nm as shown in Figure 2. The CD spectrum for GH12-M2 in buffer only showed a similar trend to GH12, with a negative peak near 235 nm. In the presence of 10% TFE, spectral flattening still occurred for both peptides with a slight increase in the MRE near 185 nm. Increasing the TFE to 20% resulted in a large difference in the CD spectrum among the peptides. Specifically, the GH12-M2 peptide in the 20% TFE solution demonstrated spectral features characteristic to the helical formation of secondary structure, including a large, positive peak near 190 nm and negative peaks near 205 and 225 nm. In contrast, the GH12 peptide alone did not show this behavior in the same 20% TFE solution. However, TFE concentrations of 40% and above elicited spectra characteristic to helical features for both the GH12 and GH12-M2 peptides.

Detailed spectral deconvolution analysis was conducted to identify the probability fractions of the relevant secondary structures including regular and disorder helices and sheets, turns, and unordered folding. The analysis revealed that GH12 and GH12-M2 have similar secondary structure fractions in the absence of TFE, with the regular helix fraction slightly larger (3% versus 0%) for the GH12 peptide. With 10% TFE in the buffer solution, the result is a similar distribution of secondary structure fractions to the peptides in buffer only. For GH12, the addition of 10% TFE to the buffer reduced the potential of the regular helix so that all the categories are within 1–2%. At solutions with 20% TFE, we observed a large difference between the secondary structure fractions for GH12 and GH12-M2, consistent with the original CD spectra results. GH12-M2 resulted in more regular and distorted helical features (38%) compared to GH12 (4%). Regular sheet and distorted sheet fractions were greater for GH12 (44%) versus GH12-M2 (15%). At high TFE concentrations (>20%), the two peptides stabilize in the fractions of their CD deconvolution. The GH12 peptide in solutions containing 40% and greater TFE exhibit a larger turn fraction (21–23%) and smaller helix fraction (37–38%) than calculated for GH12-M2 turns (16–17%) and helix fraction (42–43%).

In agreement with previous studies, GH12 exhibits helix conformations in solvents mimicking interactions at the membranes [76]. Our results are in agreement with the trends that are shared by the modified GH12 and GH12-M2. Due to significant differences in the structural analyses of CD, we did further characterization using Fourier transform infrared (FT-IR) and Raman spectroscopy.

### 3.3. Vibrational Spectroscopy

**Figure 3.**
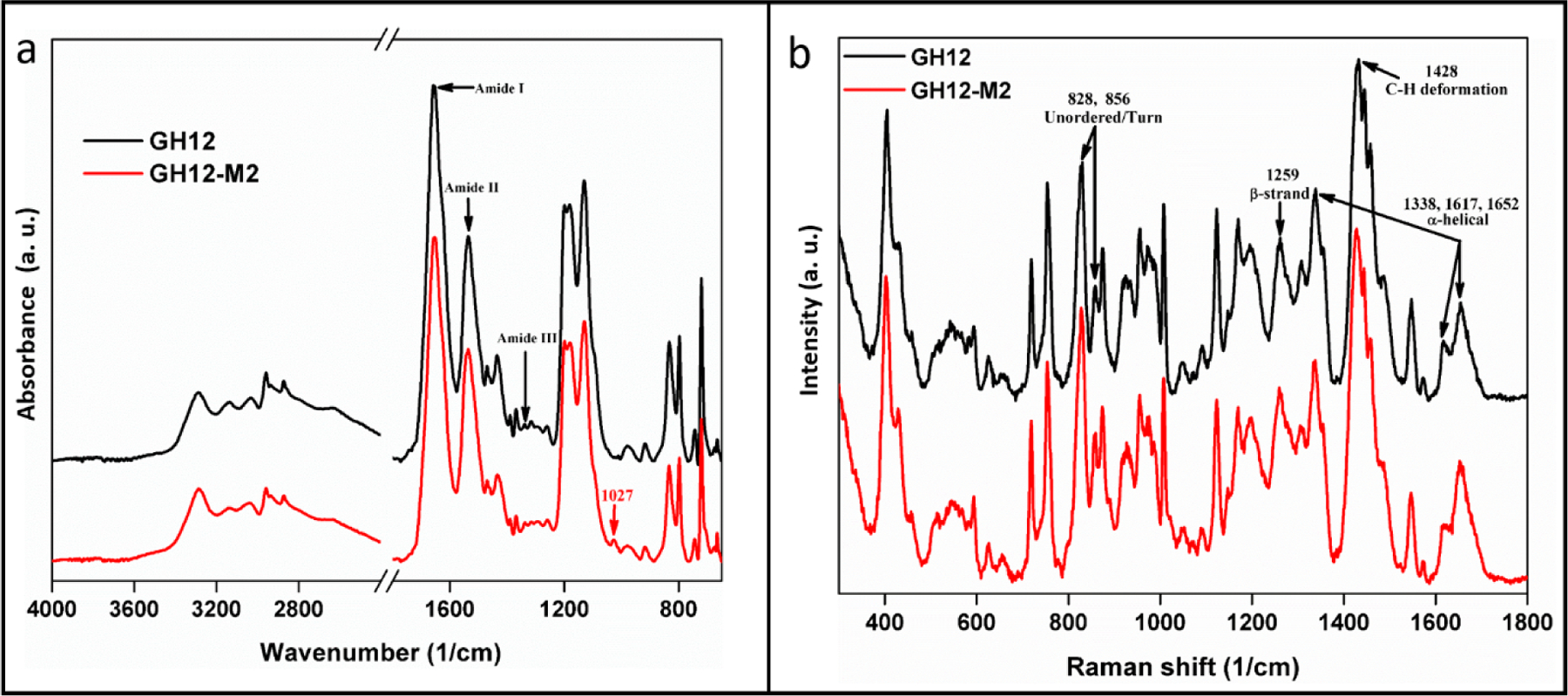
Vibration spectroscopy. (**a**) The molecular structure of the synthesized peptide was confirmed using FTIR. (**b**) GH12 to GH12-M2 peptides contained the spectral bands of 1337, 1428, and 1652 cm^−1^ characteristic of an *α*-helix conformation, the CH_2_CH_3_ deformation, and a β-strand conformation, respectively.

The secondary structure of GH12 is noted in the FT-IR spectra of GH12 and the modified derivative, GH12-M2. Peptides were compared to identify any significant spectral similarities and differences. FT-IR revealed common spectral features associated with secondary structure features for both peptides. We observed two maxima in the Amide I region at 1658 cm^*−*1^ and 1652 cm^*−*1^, a peak 1536 cm^−1^ in the amide II region, and a peak at the 1338 cm^*−*1^ in the amide III region for both peptides (Figure 3a). The characteristic Amide I peaks at 1658 cm^*−*1^ and 1652 cm^*−*1^ can be attributed to *α*-helical conformation. A peak at 1536 cm^*−*1^ is correlated to the presence of random coil. The peak at 1338 cm^*−*1^ also indicated the presence of *α*-helical structures whereas the peaks at 1259 cm^*−*1^ are assigned to unordered secondary structures and turns [77]. The only notable difference between the two peptide spectra occurred at 1027 cm^*−*1^, a spectral feature associated stretching appearing in the with CH_3_, N-H, C-N bending and stretching appearing in the spectra for GH12-M2 (Table 3).

While FTIR spectroscopy was used to indicate chemical bond absorbance, Raman spectra were studied to understand the inelastic light scattering pattern of the peptides. As a complementary technique to FTIR, data collected via Raman spectra also demonstrated high similarity between the GH12 and GH12-M2 peptides. In fact, the modification of GH12 to GH12-M2 demonstrated almost no measurable effect on its folding properties derived from Raman spectral analysis. Both peptides contained the spectral bands of 1337, 1428, and 1652 cm^*−*1^ characteristic of an *α*-helix conformation, the CH_2_CH_3_ deformation, and a β-strand conformation, respectively (Table 4) [78–82]. The intensity ratios of two bands 1617 cm^*−*1^ and 1652 cm^*−*1^ in the Amide I region for GH12 and GH12-M2 were 0.6 and 0.5, respectively (Figure 3b). The similar ratios between these two peptides indicates further agreement of the hydrogen bonding patterns measured in the Amide I region. We further explored the likely residues involved in forming helical secondary structure through computational structure flexibility.

The *α*-helix secondary structure of GH12 is a design target for functional integration of the peptide to the adhesive system [76]. The presence of *α*-helix conformation was confirmed through our vibrational spectroscopy measurements for peptides including the modified GH12. We further explored the likely residues involved in forming *α*-helix conformations through computational structure flexibility.

### 3.4. Computational Structure Flexibility

**Figure 4.**
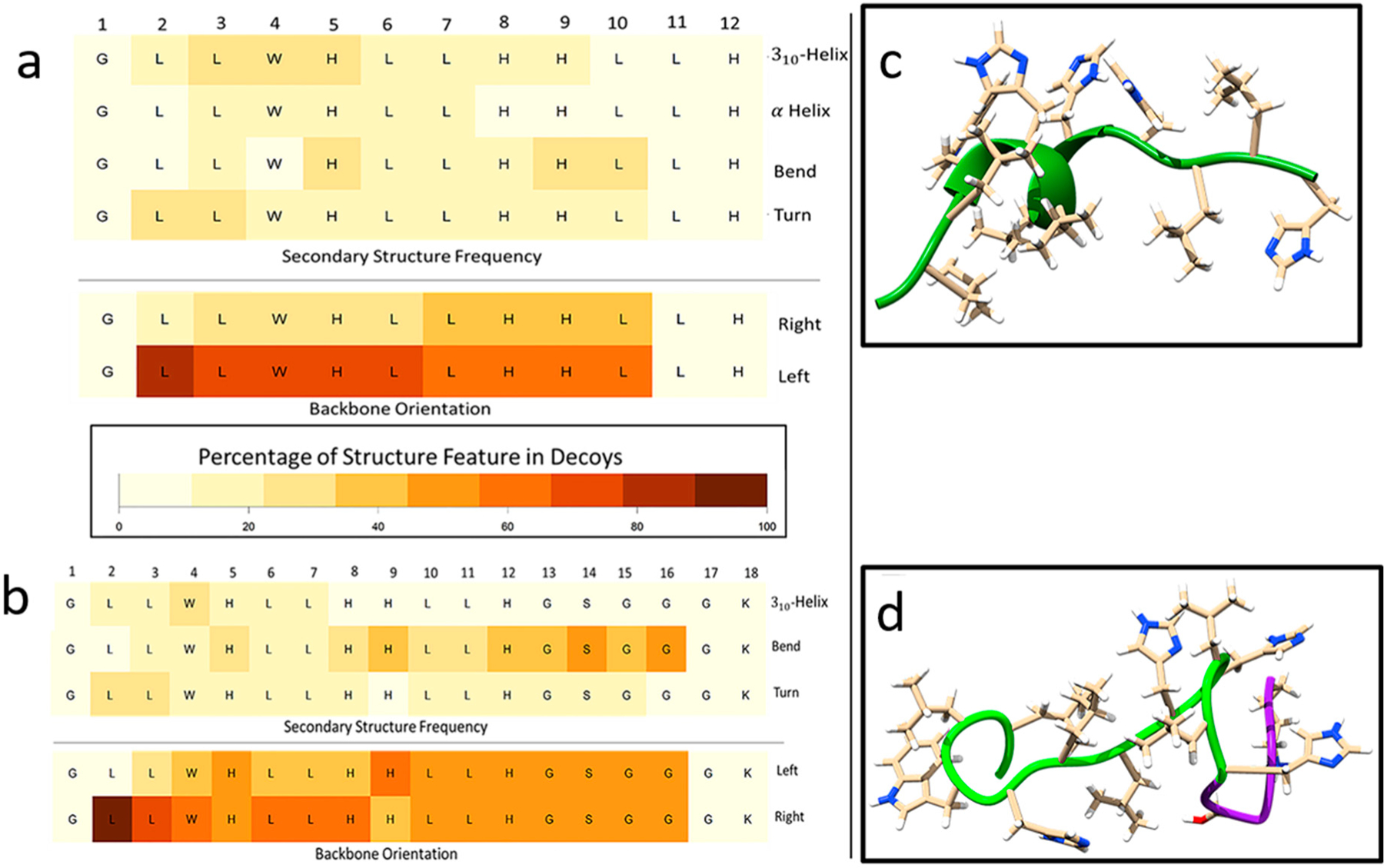
Analysis of secondary structure features predicted through PyRosetta method and quantified through DSSP(Define Seco ndary Structure of Proteins). (**a**) GH12 flexibility analysis of percentages of secondary structures (rows) occurring in the PyRosetta-generated decoys by residue calculated by DSSP. (**b**) GH12-M2 flexibility analysis of secondary structure of decoys by residue using PyRosetta and DSSP. (**c**) GH12 lowest energy score structure decoy generated through PyRosetta method. (d) GH12-M2 lowest energy structure. Green ribbon indicates the GH12 residues, and the purple ribbon indicates the modification for GH12-M2.

Secondary structures of peptide sequences can also be computationally predicted by generating structure decoys through PyRosetta. The distribution of predicted secondary structural features as a function of sequence residue for GH12 and GH12-M2 are shown in Figure 4a,b. Darker colors denote higher frequencies of the feature labeled at the right-hand side. The residue positions with the highest occurrence of alpha helix are residues 3–7 (LWHLL). The residues with the highest predicted three-helix structure frequencies are residues 3–5 (LWH). It should be noted that the first residue and last two residues cannot be labeled with these three-helix structure features because the definition used by DSSP (Define Secondary Structure of Proteins) requires the presence of adjacent residues. Bends (which are not helices) peaked at residues 5, 9, and 10. Turns, not defined as bends or helices, peaked at residues 2 and 3. The bottom two rows are defined by the direction in which the peptide backbone rotates. For about 80% of the decoys, the leucine at residue 2 is turned right, from residue 3 to 6 about 70% of decoys are turned right, and for residues 7–10 about 60% of the decoys. Figure 4c shows the helix in the GH12 peptide backbone ribbon for the lowest energy score decoy.

Figure 4d represents the GH12-M2 peptide backbone ribbon structure for the decoy with the lowest calculated energy score. No *α*-helical secondary structure was predicted for this decoy. However, the sequence modification of M2 (GSGGGK) did induce a change in the flexibility analysis results across residues 2–10 as compared to the same residues of the GH12 decoy. Even though no helix features were predicted for the lowest energy score decoy, 3_10_-helix structures were observed for about 10–20% of decoys across residues 2–7. The percentages of *α*-helix dropped below 10% for all residues of GH12-M2 compared to *α*-helix structure predictions above 10% for residues 3–7 for GH12. The GH12-M2 features of bend were calculated in residues 14 and 16 in about 50% of the decoys. The right and left turn frequencies were similar to those observed for GH12, with a 50–50% split for the spacer residues. Although the structural analysis of CD for both GH12 and our modified derivative peptide, GH12-M2, showed significant differences, FT-IR and Raman spectra analyses of both GH12 and GH12-M2 displayed only slight variations.

The difference in structure flexibility between GH12 and GH12-M2 appeared negligible in accordance with the fragment insertion method employed by PyRosetta. The decoy generation method is stochastic and therefore cannot accurately predict flexibility changes if the lowest energy structures possible are found. However, the lowest energy structure may differ from the native structure [84,85]. The parallel optimization of many decoys at once may also provide insight into the distribution of the ensemble of structures sampled by peptides in solution. Peptides are not static when floating in solution, instead they are often modeled as dynamic mechanical systems of springs and masses. constantly in motion [45,86].

The computational folding model and the circular dichroism measurements agreed with results for increasingly ordered water conditions with TFE in the buffering solution, but not for the less-ordered water conditions with phosphate-buffer only. The computational folding model used an implicit solvent method, which provided a faster structure generation process. Another advantage to the implicit solvent model was the averaging of the solvent conditions of ordered peptide states, which are closely related to the environmental conditions of the fragments when the structure was determined. This environment was not in disordered conditions such as in a dilute aqueous solution, rather fragments were obtained from crystallized structures in ordered environments.

The computational folding model estimated a higher occurrence of helical structures, while the deconvolution of the CD spectra by CD Pro predicted mainly beta-sheet structures for peptides in buffer only. However, the deconvolution method observed large increases in the helical structures with increasing amounts of 2,2,2-trifluoroethanol (TFE) in the solution. TFE is a known kosmotropic agent, increasing the order of the water near the surface of biomolecules [87]. TFE has long been used in the crystallization of proteins. The increased TFE concentrations were expected to put the peptides in an ordered, structured state [88,89]. The computational folding model held a bias toward native, ordered structures because it used fragments from crystallized structures. Whereas fragment-insertion structures generated were representative of kosmotropic agent-related peptide structures, obtained with the increasing levels of TFE. The method held a bias against generating structures representative of peptide structures with chaotropic agents such as would likely be observed with urea or guanidinium chloride. The water near bacterial surfaces is likely also highly ordered, due to the biomolecules of the bacterial membrane [76,90]. Since our structure generation method searched the portions of the structural space with ordered fragments, it may provide structures representative of bacterial surface interactions for structure–function relationships. We next explored the functionality of the peptide engineered adhesive formulations on the discs.

Incorporation of additional functionality to antimicrobial peptides benefits from detailed analyses of the structure-function relationships on the resulting peptides as discussed in our group’s recent papers on antimicrobial peptides based coatings or films [43,44,54,91]. In this study design, we investigated if residues of the spacer domain added for the conjugation to the polymer affect the peptide folding, which in turn may have an adverse effect on the antimicrobial property. We observed similar circular dichroism spectra for GH12 and GH12-M2 in Figure 2. We also obtained similar analyses for both peptides using FTIR and Raman spectra as shown in Figure 3. Almost no difference in structural flexibility in the peptide models were observed (Figure 4). The similarity of observed and computationally predicted structure between GH12 and GH12-M2 gives an indication that GH12-M2 is a good candidate to conjugate to the resin without losing its activity.

### 3.5. Bacterial Viability on Treated Discs

We first explored if the resin formulations have any antibacterial effects, we prepared discs using both commercial analogue and ε-pL polymerized discs. The discs were soaked in water overnight for a baseline control. Neither formulation exhibited any innate antibacterial activity against *S. mutans*. We next tested the antibacterial activity on the discs soaked in antimicrobial peptide solutions across a range of concentrations up to 50× the minimum inhibitory concentration (MIC) (1560 μg/mL). The ε-pL discs soaked with peptide solutions exhibited significant antibacterial activity against *S. mutans*. Complete bactericidal efficacy was observed for ε-pL discs soaked in GH12-M2 peptide at 25*×* MIC value (780 μg/mL). Near complete reduction in *S. mutans* growth was observed at 12.5*×* MIC (390 μg/mL), however antibacterial activity was not statistically significant at concentrations of 6.25*×* MIC (195 μg/mL) and below for the GH12-M2 peptide. The positive control studies included discs that are soaked in chlorhexidine (CHX), where robust antimicrobial property was observed for both discs, but the CHX showed higher growth inhibition in the commercial analogue discs as compared to the ε-pL formulation discs. CHX-soaked discs exhibited complete bactericidal efficacy against *S. mutans* at 25× and 12.5× MIC concentration for each disc formulation. While, the CHX was effective in both disc formulations at different levels, GH12-M2 peptide demonstrated antibacterial activity in disc formulations featuring ε-pL. These results indicate a critical dependence on the adhesive formulation to incorporate functional peptides.

**Figure 5.**
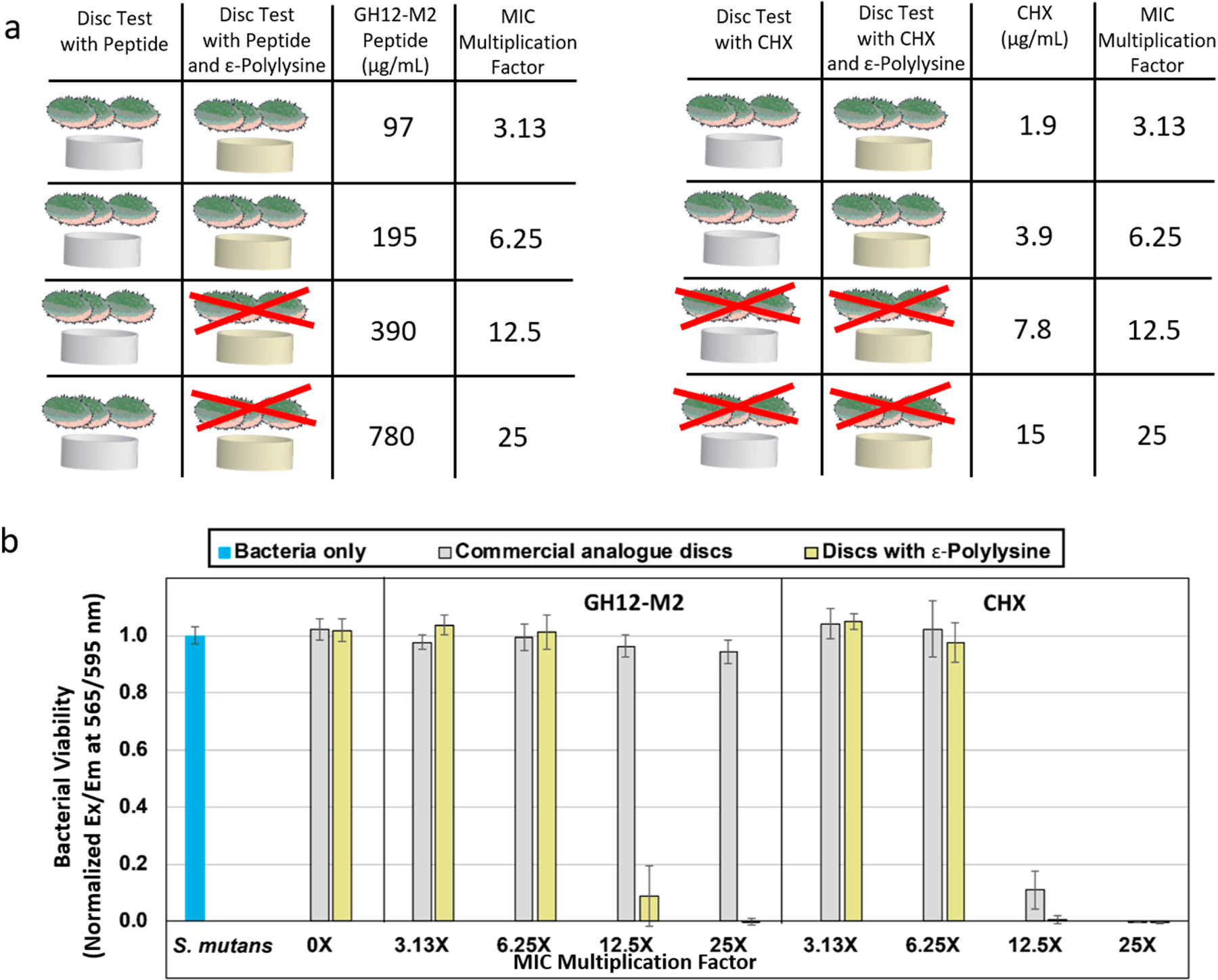
(**a**) Schematic of adhesive system samples starting with commercial analogue. Experiments included the addition of GH12-M2 antimicrobial peptide onto the discs with ε-polylysine to see bacterial inhibition. As a positive control for antimicrobial activity, chlorhexidine gluconate was added at a multiple of its measured MIC value. 12.5× or greater concentration of CHX was sufficient for bacterial inhibition with or without ε-polylysine. (**b**) *S. mutans* UA159 growth/viability based on AlamarBlue assay on dental adhesive discs soaked with either an antimicrobial peptide or a chlorhexidine aqueous solution as a multiple of minimum inhibitory concentration.

Though the ε-pL discs coupled with GH12-M2 peptide demonstrated bactericidal efficacy at concentrations of 390 μg/mL and 780 μg/mL, commercial analogue discs coupled with peptide at these concentrations did not show any significant bactericidal efficacy. Engineering the peptide to the adhesive formulation has a synergetic antibacterial effect when incorporated into ε-pL adhesive system, while commercial analogue for the same peptide concentrations did not show similar synergetic effect (Figure 5). The results demonstrate the importance of biocompatible adhesive system to induce antimicrobial effects through novel antimicrobial agents. An increased occurrence of affinity and non-specific interactions of the GH12-M2 peptide with commercial analogue resin discs was noted based upon the observed reduction in bacterial viability at peptide concentrations of 390 μg/mL and 780 μg/mL, as seen in Figure 5b. The hydropathy of the resin with ε-Polylysine is hydrophilic and highly positively charged, unlike bacterial membranes. Since the commercial analogue is more hydrophobic, the peptide is likely adsorbing to the resin with the similar hydrophobic interactions, such as hydrogen bonding and van der Waal forces, that occur in the bacterial membrane [92].

The delivery mechanism of the antimicrobial agents, either the peptide or the CHX, is based on the non-specific adsorption of the agent to the resin. The antimicrobial agents are coupled to the resin through adsorption overnight before the bacteria are added to the resin material. Unbound agent is also rinsed off before bacteria are added. Therefore, only the adsorbed agent is available for any bactericidal effect. However, the resin must act as a delivery system so that the adsorbed agent is active against the introduced bacteria. The non-specific adsorption of the peptide to the commercial analogue resin appears to be more stable than the potential interactions taking place between either agent is active against the introduced bacteria. The non-specific adsorption of the peptide to the commercial analogue resin appears to be more stable than the potential interactions taking place between either the peptide and the ε-Polylysine disc or the CHX and both resin discs. The stability of this non-specific adsorption for antimicrobial peptides on polymeric materials are considered as a result of the similarities between polymers and bacterial membranes [92]. Non-specific interactions of antimicrobial peptides with polymers that mimic bacteria membranes limit their availability for antibacterial efficacy. Thus, there is a challenge to overcome these non-specific interactions. Our approach is to provide an adequate orientation of the peptide away from the resin through coupling the antimicrobial peptide to the resin with a spacer domain in between the peptide and the resin. The spacer domain is always anchored to the resin. Anchoring the peptides to the resin minimizes the interactions between peptides. Several studies including ours have shown that spacer domains provides flexibility for peptides to keep their active conformation while they interact with the surfaces, which improves their biological function [43,54,93]. For example, a rigid spacer was shown to improve the antibacterial activity of a peptide compared to a flexible spacer of the same length [43]. In this work it was noted that the addition of a spacer sequence, flexible or rigid, allowed greater functional activity of the peptides coupled to the resin, when compared to respective peptides without a spacer [93]. Overall spacer design approach which was inspired by the domain linker sequences observed in multi-domain proteins, provided an active orientation for the antimicrobial peptide when coupled to the adhesive formulation. [94,95].

## 4. Conclusions

A dental adhesive formulation with ε-Polylysine was designed to incorporate antimicrobial peptide to target *S. mutans.* The choice of ε-Polylysine as dental adhesive was inspired due to its non-toxic nature. Our studies included design of peptides with different modification for their effective coupling to adhesive formulations. The peptides were first designed and tested for their antimicrobial activity in solution. We then performed complementary structural characterization and computational modeling methods to understand the effect of the helical conformations of GH12, which is a design target for the synthetic peptide. As we modified the peptide, structural properties of the peptide were investigated to relate its antimicrobial functionality prior to designing peptide coupled adhesive formulations. The peptide conformation was depicted at the solid interfaces both computationally and experimentally using a kosmotropic agent, TFE. At higher TFE concentrations, peptides demonstrated spectral features characteristic to the helical formation of secondary structure. The *α*-helix secondary structure of the modified GH12 was next confirmed through vibrational spectroscopy measurements. Building upon the expected structures of the peptides that can provide functionality, we finally explored the antimicrobial property of the peptide-adhesive formulations, i.e., ε-Polylysine and commercial analogue adhesive system. The peptide adhesive formulation demonstrated significant antimicrobial activity against *S. mutans* when applied to the discs that are made of ε-Polylysine. Interestingly, the antimicrobial property of the commercial analogue adhesive system when soaked with antimicrobial peptide was limited. These results indicate the critical importance of presenting biofunctionality when developing adhesive formulations coupled with molecular novel agents. Our results demonstrate that bio-enabled dental adhesive system provides bacterial inhibition to be employed at the adhesive/dentin interface.

Antimicrobial peptides offer a promising solution to antibiotic resistance, staining, and mucosal irritation issues related to existing approaches. Our results demonstrate that antimicrobial peptides can be coupled with dental adhesive formulations using a simple, one-step soaking process. The peptide structure–function analyses indicate that peptides can also be incorporated with additional functional groups that will allow them to conjugate to different monomer/polymer combinations. Integrating an antibacterial activity through a bio-active molecule conjugation within an adhesive provides a path for integrating various biofunctions at the interface of hybrid materials. This methodology may also be applied to integrate bio-active molecular activity into polymeric materials ranging from medical to diverse engineering applications.

## Supplementary Material

Supplementary Material

## Figures and Tables

**Table 1.** Commercial analogue synthesis and ε-pL adhesive synthesis. HEMA: 2-hydroxyethylmethacrylate; TEGDMA: triethylene glycol dimethacrylate; MPC: 2-methacryloyloxyethyl phosphorylcholine.

Component	Commercial Analogue (wt%)	ε-Polylysine (wt%)
HEMA	64	59
TEGDMA	10	10
MPC	10	10
ε-Polylysine	/	5
Water	14	14
Photo-initiators	2	2
Total	100	100

Photo-initiators: Camphorquinone (CQ) 0.5 wt%, ethyl-4-(dimethylamino) benzoate (EDMAB) 0.5 wt%, and diphenyliodonium hexafluorophosphate (DPIHP) 1.0 wt%.

**Table 2.** Properties of antimicrobial peptides, i.e., GH12, and its modifications, and ε-polylysine.

Peptide	Sequence	*S. Mutans* IC_50_ (μg/mL)	*S. Mutans* MIC (μg/mL)
GH12-NH_2_	GLLWHLLHHLLH (-NH_2_ )	-	6.7 *
GH12	GLLWHLLHHLLH (-COOH)	11.6	31.3
GH12-M1	K_GGGSG_GLLWHLLHHLLH	17.1	31.3
GH12-M2	GLLWHLLHHLLH_GSGGG_K	12.8	31.3
ε-Polylysine	(ε-Lysme)_n_	371.3	125

* As reported by Tu, et. al., in a 2016 study.

**Table 3.** FTIR spectroscopy of GH12 and GH12-M2.

Peak Region Name	Peak (cm^−1^)	Peptides
Amide I	1658,1652 (α-helical structures)	GH12 (twin), GH12-M2
Amide II	1536	GH12, GH12-M2
Amide III	1338 (α-Helical structures)	GH12, GH12-M2
Amide III	1259 (Unordered/turn)	GH12, GH12-M2
CH3,N-H,C-N stretching-bending [83]	1027	GH12-M2

**Table 4.** Raman spectra of GH12 and GH12-M2 by Diode785.

Shift Peak Structure	Shift Peak (cm^−1^)	Peptides
α-Helical structures	1617, 1652	GH12 (ratio: 0.6), GH12-M2 (ratio: 0.5)
CH2CH3 deformations	1428	GH12, GH12-M2
β-Strand conformation	1259	GH12, GH12-M2
Unordered/turn	856, 828	GH12, GH12-M2
