# Supplementary material for: Peptide Mediated Antimicrobial Dental Adhesive System
Source: Appl Sci (Basel). Author manuscript; Available in PMC 2021 Feb 3. (PMC7857482; doi:10.3390/app9030557)
Supplement: Supplementary Material [file NIHMS1015942-supplement-Supplementary_Material.pdf]

Supplementary Material

Peptide Mediated Antimicrobial Dental Adhesive System

Sheng-Xue Xie [1], Kyle Boone [1,2], Sarah Kay VanOosten [1,2], Esra Yuca [1,3], Linyong Song [1], Xueping Ge [1], Qiang Ye [1], Paulette Spencer [1,2,4], and Candan Tamerler [1,2,4,*]

[1] Institute for Bioengineering Research, University of Kansas, 1530 W. 15th St., Lawrence, KS 66045, USA; sxie@ku.edu (S.X.X.); k097b443@ku.edu (K.B.); sarah.vanoosten@ku.edu (S.K.V.); e782y752@ku.edu (E.Y.); LeonSong@ku.edu (L.S.); xpge718@gmail.com (X.G.); yeq@ku.edu (Q.Y.); pspencer@ku.edu (P.S.)

[2] Bioengineering Program, 1530 W. 15th St., University of Kansas, Lawrence, KS 66045, USA

[3] Department of Molecular Biology and Genetics, Yildiz Technical University, 34210, Istanbul, Turkey; eyuca@yildiz.edu.tr (E.Y.)

[4] Department of Mechanical Engineering, 1530 W. 15th St., University of Kansas, Lawrence, KS 66045, USA

* Correspondence: ctamerler@ku.edu

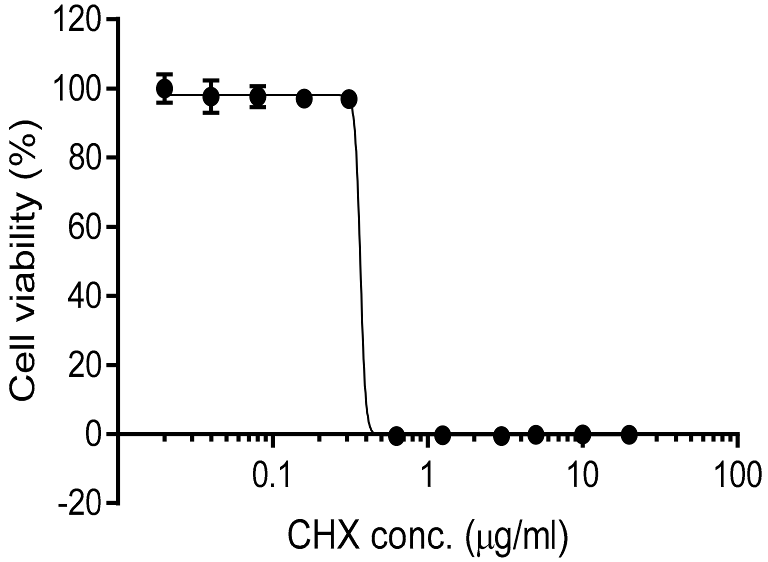

Figure S1. MIC (0.63 µg/ml) and IC_{50} (0.37 µg/ml) of CHX against *S. mutans* (R^2 = 1.0).

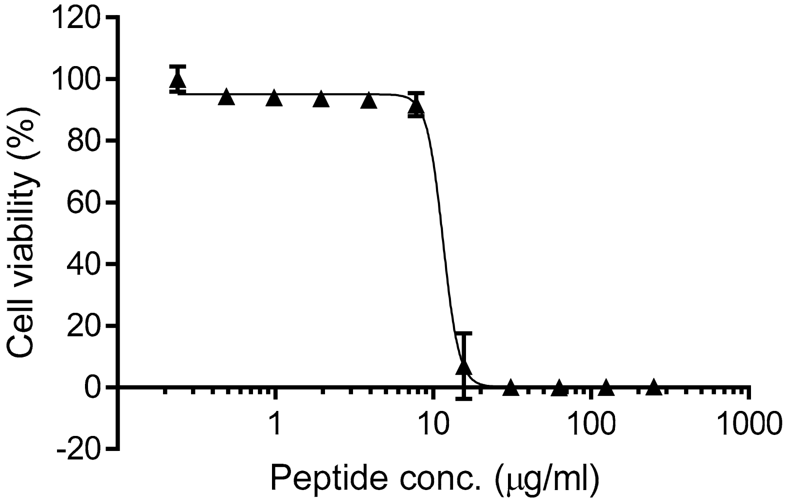

Figure S2. MIC (31.3 μg/ml) and IC_{50} (11.55 μg/ml) of GH12 against *S. mutans* ($R^2 = 0.99$).

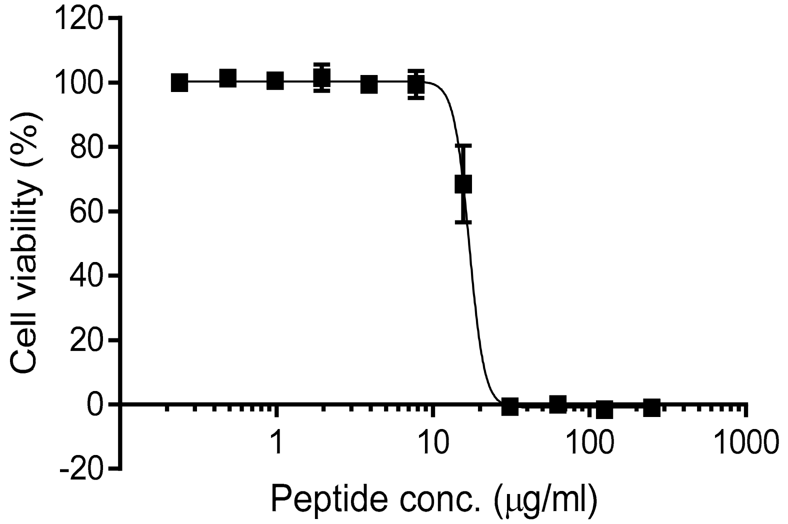

Figure S3. MIC (31.3 μg/ml) and IC_{50} (17.05 μg/ml) of GH12-M1 against *S. mutans* ($R^2 = 0.99$).

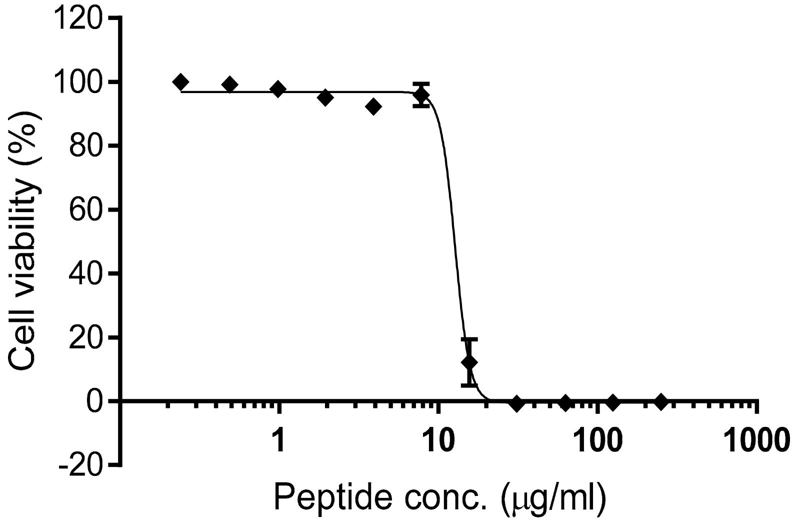

Figure S4. MIC (31.3 μg/ml) and IC_{50} (12.75 μg/ml) of GH12-M1 against *S. mutans* (R^2 = 1.0).

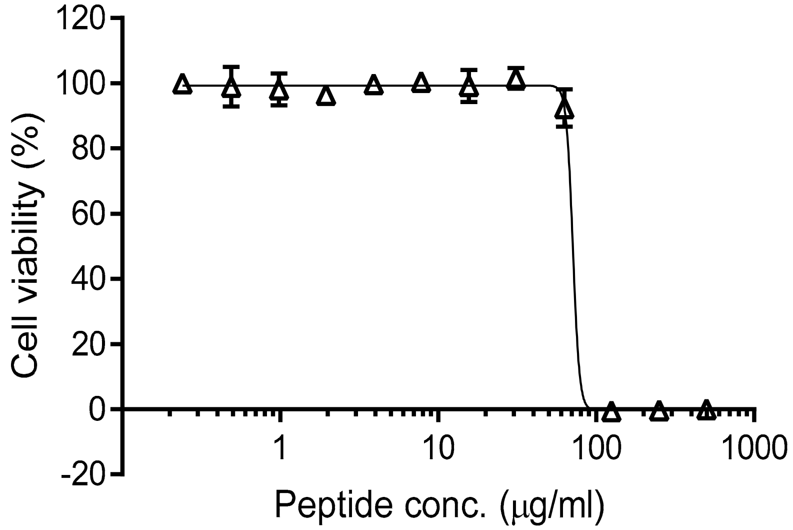

Figure S5. MIC (125 μg/ml) and IC_{50} (71.27 μg/ml) of ε-Polylysine against *S. mutans* (R^2 = 1.0).
